# Template Synthesis of Co(II), Ni(II) and Cu(II) Complexes Derived From Oxamide Ligand and the Reactivity of Cu(II) Complex Towards Human Serum Albumin

**DOI:** 10.1155/S1565363304000147

**Published:** 2004

**Authors:** Bhawana Mohani, Farukh Arjmand

**Affiliations:** Department of Chemistry Aligarh Muslim University Aligarh 202002 India

## Abstract

A new oxamide ligand 2,2'-(oxalydimino)bis(diacetic acid)[C_10_H_11_O_10_N_2_],[L] has been synthesized by the condensation reaction of Iminodiacetic acid and Diethyloxalate. This ligand [L] was further allowed to interact with triethylene-tetraamine metal complexes [C_16_H_26_N_6_O_8_M]Cl_2_ (where M=Co^11^, Ni^11^ and Cu^11^) to yield the new N4 macrocyclic complexes 3, 3', 6, 6' tetraazadodeca 1-1' diimino N N tetraacetic acid M) chloride ([C_16_H_26_N_6_O_8_Co]Cl_2_, [C_16_H_26_N_6_O_8_Ni]Cl_2_ and [C_16_H_26_N_6_O_8_Cu]Cl_2_). These complexes were characterized by elemental analyses, i.r., n.m.r., e.p.r, and u.v.-vis spectroscopy. All the complexes show square planar geometry and are ionic in nature. The kinetic studies of the Cu(lI) complex were ascertained spectrophotometrically by observing the absorbance changes in presence of protein Human Serum Albumin (HSA) in phosphate buffer at different pH's at 30^0^C. The absorbance changes were monitored at 278 nm (λ_max_ of HSA) with respect to time and pseudo-first-order rate constants, kobs, were obtained from the slope of the straight line using the least squares regression method. The electrochemical behaviour of the Cu(ll) complex was monitored by cyclic voltammetry in a phosphate buffer. The E_p_ values-0.730 and-0.560 V respectively, were obtained at the scan rate of 0.1 Vs^-1^. The interaction of the Cu(II) complex with the HSA was studied at the same scan rate, which reveals weak binding as the E^0^ values do not shift considerably. The cyclic voltammogram of the Cu(II) complex bound to HSA was recorded at different pH's also (6.5 to 7.4). The pH-rate profile data reveals that the reactions are pH dependent.


### Template Synthesis of Co(II), Ni(II) and Cu(II) Complexes Derived From Oxamide Ligand and the Reactivity of Cu(II) Complex Towards Human Serum Albumin.

Bhawana Mohani and Farukh Arjmand*.

*Department of Chemistry, Aligarh Muslim University, Aligarh-202002, India*

### ABSTRACT

A new oxamide ligand 2,2'-(oxalydimino)bis(diacetic acid)[$C_{10}H_{12}O_{10}N_2$],[L] has been synthesized by the condensation reaction of Iminodiacetic acid and Diethyloxalate. This ligand [L] was further allowed to interact with triethylene-tetraamine metal complexes [$C_{16}H_{26}N_6O_8M$]$Cl_2$ (where M = $Co^{II}$, $Ni^{II}$ and $Cu^{II}$) to yield the new $N_4$ macrocyclic complexes 3, 3', 6, 6' tetraazadodeca 1-1' diimino N N tetraacetic acid $M_{(II)}$ chloride ([$C_{16}H_{26}N_6O_8Co$]$Cl_2$, [$C_{16}H_{26}N_6O_8Ni$]$Cl_2$ and [$C_{16}H_{26}N_6O_8Cu$]$Cl_2$). These complexes were characterized by elemental analyses, i.r., n.m.r., e.p.r. and u.v.-vis spectroscopy. All the complexes show square planar geometry and are ionic in nature. The kinetic studies of the Cu(II) complex were ascertained spectrophotometrically by observing the absorbance changes in presence of protein Human Serum Albumin (HSA) in phosphate buffer at different pH's at 30$^0$C. The absorbance changes were monitored at 278 nm ($\lambda_{max}$ of HSA) with respect to time and pseudo-first-order rate constants, $k_{obs}$, were obtained from the slope of the straight line using the least squares regression method. The electrochemical behaviour of the Cu(II) complex was monitored by cyclic voltammetry in a phosphate buffer. The $E_p$ values –0.730 and –0.560 V respectively, were obtained at the scan rate of 0.1Vs$^{-1}$. The interaction of the Cu(II) complex with the HSA was studied at the same scan rate, which reveals weak binding as the $E^0$ values do not shift considerably. The cyclic voltammogram of the Cu(II) complex bound to HSA was recorded at different pH's also (6.5 to 7.4). The pH-rate profile data reveals that the reactions are pH dependent.

**Keywords:** Oxamide ligand; Cu(II)complex; Human Serum Albumin Binding; Kinetics; Electrochemistry

### 1. INTRODUCTION

Transition metal ions Cu(II), Ni(II) and Zn(II) bind to imidazole side chain of surface exposed histidines of proteins /1-3/. However, the binding to protein active site domain is stereospecific /4/. Much emphasis has been laid on the design of compounds with increased affinity for a molecular target due to wide applications in pharmaceutical industries /5-8/. Protein-ligand complexes were designed to maximize the interactions between a protein and a small molecule or, alternatively, alter the chemical properties viz. solubility without disrupting binding. The same approach has been employed to design compounds with decreased affinity for HSA. Compounds having reduced affinity for serum albumin can prove as potent drugs with significantly lower dosage levels and improved in vivo tolerance /9/.

The Human Serum Albumin (HSA) are the major soluble protein constituents of the circulatory system, hence involved in the transport, distribution and metabolism of many endogenous ligands such as fatty acids, bilirubin, amino acids, metals etc and numerous pharmaceuticals as well /10-13/. The main function of this protein is as carrier and recent report on HSA reveals the binding studies of Penicillins to HSA.[14,15]. HSA's have six different lysine residues and one of these groups, Lys-199 is the most probable binding site for compounds /16/ (Fig. 1).

To study the specific interactions of HSA-metal complex having reduced albumin binding, herein we report the design and synthesis of novel metal complexes derived from oxamidediiminodiacetic and triethylene tetramine. Cyclic voltammetry and kinetic experiments were performed in phosphate buffer at different pH's to ascertain the binding mode of the Cu(II) complex of oxamidediiminodiacetic acid to HSA. In our earlier work /17/, we have studied the effect of the Co(II) complex of 1,2bis(sulphapyridyl)oxamide with Bovine Milk Casein (BMC.)

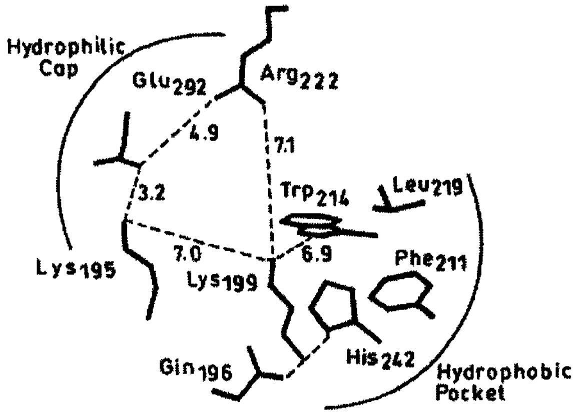

**Fig. 1:** The environment of the $Lys_{199}$ binding site in HSA as obtained directly from X-ray refinement. Distances in $A^0$.

### 2. EXPERIMENTAL

### 2.1. Reagents

Iminodiacetic acid, diethyl oxalate (Merck), $CoCl_2.6H_2O$, $NiCl_2.6H_2O$ and $CuCl_2.2H_2O$ and triethylene tetramine (BDH), were used as received.

### 2.2. Synthesis of the ligand 2,2'-(oxalydimino)bis(diacetic acid)[$C_{10}H_{12}O_{10}N_2$],[L]

Iminodiacetic acid (5 gm) in water (10 $cm^3$) was added to diethyloxalate (1.35 gm) in a 2:1 molar ratio. The above solution was refluxed for *ca.* 6 h and then conc. HCl (6$cm^3$) was added dropwise with constant stirring. The resulting solution was refluxed for *ca.* 48 h and allowed to cool overnight in refrigerator. A white amorphous product was obtained, filtered, washed thoroughly with $Et_2O$ and dried in vacuo (3.4 g, Yield 68%), M.p. 80+ 3°C. Anal. Calc. for $C_{10}H_{12}O_{10}N_2$ Found%:C, 37.71; H, 3.88; N, 8.86. Found: C, 37.50; H, 3.75; N, 8.75%. IR (KBr disc, $cm^{-1}$): 1577 and 1498 v($CO_2^-$); 1080 v(C-C); 1300 v(C-N); and1680 v(C = O). $^1H$ NMR. ($D_2O$ ppm) 11.01 COOH; 3.2-3. (-$CH_2$-).$^{13}C$ NMR 158.59 (C = O); 167.23 ($CO_2^-$); 30.96-27.54 (-$CH_2$).

### 2.3 Synthesis of the complex 3, 3', 6, 6' tetraazadodeca 1-1' diimino N N tetraacetic acid copper(II) chloride [$C_{16}H_{26}N_6O_8Cu$]$Cl_2$,

The ligand L (0.640 gm) in EtOH (50 $cm^3$) was refluxed with Cu(II) complex of triethylenetetraamine (0.538 gm) in MeOH, which was prepared by the earlier reported method /18/. The resulting solution was boiled under reflux for *ca.* 6h. The reaction mixture was then allowed to stand in refrigerator for *ca.* 24 h. Dark blue coloured powder was obtained, which was filtered off, washed with $Et_2O$ and dried in vacuo (0.36 g, yield 56 %). Scheme 1. Anal. Calc. for ($C_{16}H_{26}N_6O_8Cu$)$Cl_2$ Found%: C, 34.46; H, 4.93; N, 15.05; Calc.%: C, 34.02; H, 4.60; N, 14.88. IR: (KBr disc,$cm^{-1}$) 1576 and 1490 v($CO_2^-$); 1078 v(C-C); 1306 v(C-N); 1580-1610 v(C = N) and 470 v(M-N).

A similar procedure was also adopted for the $Co^{II}$ and $Ni^{II}$ chloride complexes.

Anal. Calc. for ($C_{16}H_{26}N_6O_8Co$)$Cl_2$ 0.30 g, yield 47%, Found%: C, 34.34; H, 4.68; N, 15.16; Calc.%: C, 34.28; H, 4.64; N, 15.00 IR (KBr disc,$cm^{-1}$) 1570 and 1494 v($CO_2^-$); 1072 v(C-C); 1303 v(C-N); 1588-1620 v(C = N) and 468 v(M-N).

Anal. Calc. for ($C_{16}H_{26}N_6O_8Ni$)$Cl_2$ 0.32 g, yield 50%, Found%: C, 34.22; H, 4.70; N, 15.16; Calc.%: C, 34.31; H, 4.64; N, 15.01. IR (KBr disc,$cm^{-1}$) 1572 and 1491 v($CO_2^-$); 1070 v(C-C); 1308 v(C-N); 1581-1612 v(C = N) and 471 v(M-N). 11.01 (COOH); 3.70- 3.90 (-$CH_2$-); 5.60 (NH). $^{13}C$ NMR (ppm): 174.21 (C = N); 167.23 ($CO_2^-$); 30.96-27.54 (-$CH_2$).

### *Other physical measurements*

Microanalyses of the complexes were obtained on a Carlo Erba Analyzer Model 1106. Molar conductances were measured at room temperature on a Digisun Electronic Conductivity Bridge. I.r. spectra (400-4000 $cm^{-1}$) were recorded on a Carl Ziess Specord M-80 spectrophotometer in KBr. $^1H$ and $^{13}C$ n.m.r. spectra were recorded on an amx-500 instrument. Cyclic voltammetry was carried out on a CH instrument electrochemical analyzer. Phosphate buffer were employed for the cyclic voltammetric (c.v.) studies with 0.4 M $KNO_3$ as a supporting electrolyte. A three-electrode configuration was used comprising a Pt disk working electrode, Pt wire counter electrode and Ag/AgCl reference electrode. Experiments were carried out at room temperature.

Kinetic experiments were performed under pseudo-first order conditions using a Systronic 119 spectrophotometer.

### 3. RESULTS AND DISCUSSION

### 3.1 EPR spectra

The e.p.r. spectra of the Cu(II) complex, recorded at 30°C, shows a signal for $g_{\parallel}$ and $g_{\perp}$ at 2.16 and 2.05 respectively. The existence of $g_{\parallel} > g_{\perp}$ suggests that $d_{x^2-y^2}$ is the ground state for the $d^9$ [$Cu^{2+}$] configuration *i.e.* ($eg^4$) $(a_1g)^2$ $(b_2g)^2$ $(b_1g)^1$ /19/. The g values are related to the axial symmetry parameter G by the expression G = ($g_{\parallel}$ - 2/ $g_{\perp}$- 2) /20/. The g value measures the extent of exchange interaction between the copper centers in polycrystalline solids. If G < 4, considerable exchange interaction occurs and if G > 4, exchange interaction is negligible. In the present case, G appears to be greater than 4, which shows that exchange interactions are absent in the complex. The presence of $g_{\parallel} > g_{\perp}$ in the spectrum of the Cu(II) complex is an authentic evidence for square planar geometry around the copper(II) atom /21/.

### 3.2. Electronic spectra

The electronic spectra of the Cu(II) complex exhibit two bands at 32,573, and 16,666 $cm^{-1}$. The assignments of the spectral band at 16,666 attributed to $^2B_{1g} \rightarrow {}^2E_{1g}$ and $^2B_{1g} \rightarrow {}^2A_{1g}$ $cm^{-1}$ transitions /22/ and the other band at 32,573 $cm^{-1}$ correspond to a ligand to metal charge transfer band (LMCT) respectively /23/ (Fig. 2). The presence of this charge transfer band in visible region has been attributed to a square planar arrangement around the Cu(II) center /24/.

The Co(II) complex also exhibits intense bands in high energy region 33,220-26,525 $cm^{-1}$ which are assigned to charge transfer M → L bands. The two low energy bands are identified at 20,080 and 19,521 $cm^{-1}$ due to $^2A_{1g} \rightarrow {}^2E_g$ transition indicating square planar geometry /25/. The electronic spectra of Ni(II) complex also shows two bands at 26,455 and 16,886 $cm^{-1}$. The band at 26,455 $cm^{-1}$ has been assigned to $^1A_{1g} \rightarrow B_{1g}$ transition /26,27/. A single intense d-d characteristic band at 16,886 $cm^{-1}$ is attributed to diamagnetic square planar Ni(II) complex /28-30/.

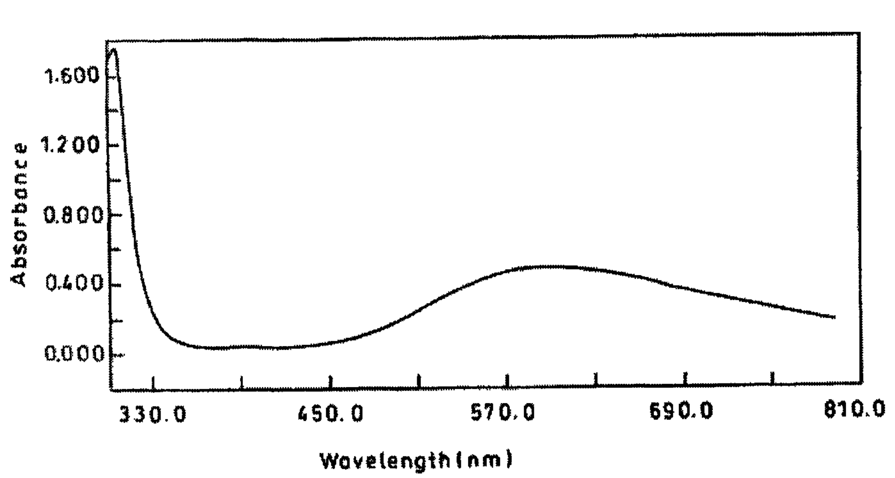

**Fig. 2:** The representative electronic spectra of Cu(II) complex.

### 3.3. Redox behaviour

The redox behaviour of the Cu(II) complex was studied by cyclic voltammetric measurement in phosphate buffer. The cyclic voltammogram of Cu(II) complex exhibits one quasi-reversible redox couple corresponding to the $Cu^{II}/Cu^{I}$ with $E_p$ values -0.730 and –0.560V respectively at a scan rate of 0.1 $Vs^{-1}$ [Fig.3a]. For this couple, the difference between cathodic and anodic potential $\Delta E_p$ is of the order 128 mV and the $I_{pa}/I_{pc}$ value is less than one. At different scan rates [Fig. 3b], there is no major change in $E_p$ and $E_{1/2}$ values, clearly indicating that $E_p$ is independent of scan rate . For a reversible/quasi-reversible wave $E_p$ is independent of scan rate and $I_p$ is proportional to $v^{1/2}$ /31, 32/. On interaction of the Cu(II) complex with HSA, there is a slight shift in $E_p$ values of –0.799 and –0.541 V respectively, at the same scan rate [Fig. 4a], suggesting the binding of HSA with the Cu(II) complex but as the shifts in formal potential of the complex are not so significant (Fig. 4b), therefore it is inferred that the Cu(II) complex exhibits reduced binding for serum albumin.

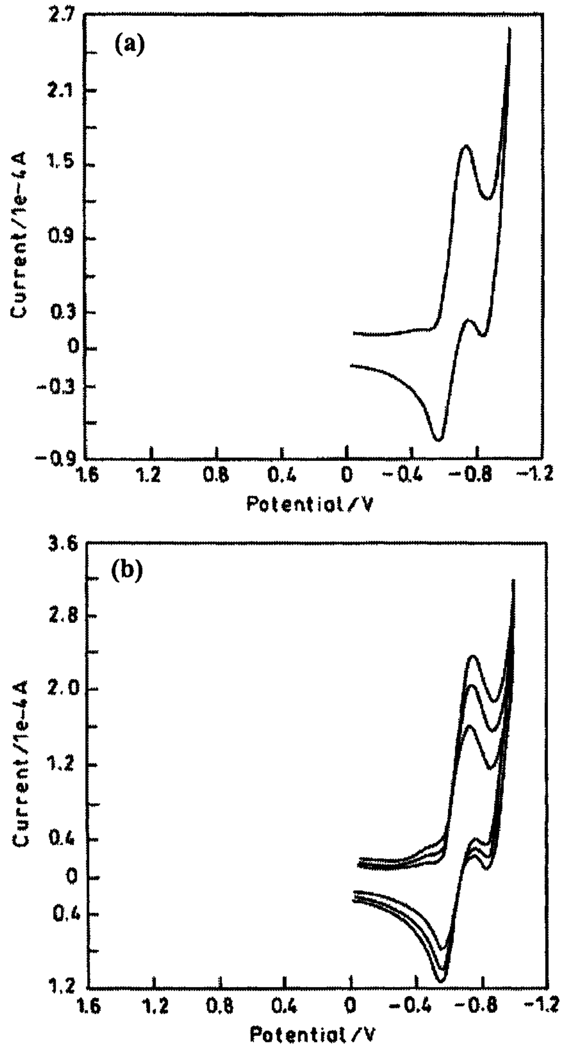

**Fig. 3:** (a) Cyclic voltammogram of Cu(II) complex in phosphate buffer at 30°C at a scan rate of 0.1 $Vs^{-1}$.
(b) Cyclic voltammogram of Cu(II) complex in phosphate buffer at 30°C at different scan rates viz: 0.1, 0.2 and 0.3 $Vs^{-1}$.

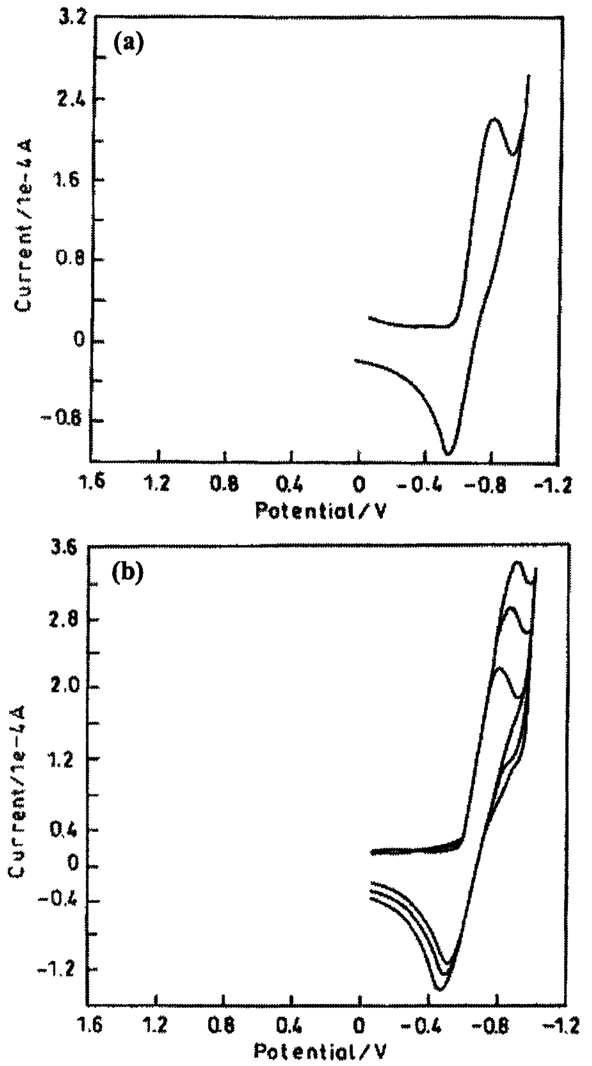

**Fig. 4:** **(a)** Cyclic voltammogram of Cu(II) complex bound with HSA in phosphate buffer at 30°C at a scan rate of 0.1 $Vs^{-1}$.

**(b)** Cyclic voltammogram of Cu(II) complex bound with HSA in phosphate buffer at 30°C at different scan rates viz: 0.1, 0.2 and 0.3 $Vs^{-1}$.

The cyclic voltammogram of the Cu(II) complex bound to HSA was recorded at different pH's (6.0-7.4). Figure 5 shows the plots of $E_p$ versus pH's. At lower pH 6.0, E° values are lowest due to partial involvement of imidazole nitrogen of HSA. The pH dependence in histidine-metal interactions has also been demonstrated earlier /33/.

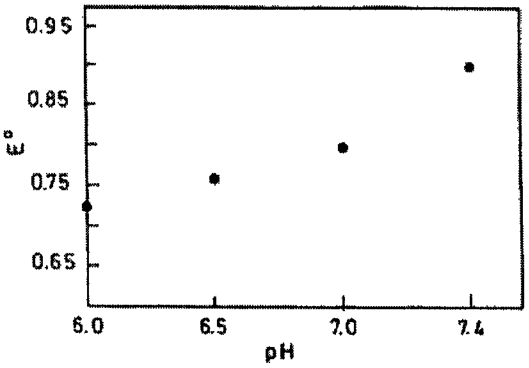

**Fig. 5:** pH dependent plot of E° value of Cu(II) complex bound with HSA at 30°C at a scan rate of 0.1 $Vs^{-1}$.

### 3.4. Protein HSA binding studies

The u.v./vis. spectrum of Cu(II) complex in phosphate buffer reveals a M-L charge transfer band at 307 nm and a d-d transition at 662 nm.

Interaction of Human Serum Albumin with the Cu(II) complex was studied at $\lambda_{max}$ of HSA (278 nm) under pseudo first order conditions. The absorbance changes with varying concentrations (c = 1 x $10^{-5}$ to 5 x $10^{-5}$ mol $dm^{-3}$) of HSA in buffer solution were recorded at fixed concentration of the Cu(II) complex ( c = 0.5 x $10^{-5}$ mol $dm^{-3}$) at the different time intervals at 30°C . After the addition of HSA at the different time intervals (Fig. 6), there is a steep decrease in absorption intensity indicative of hypochromicity; however, no band shift has been observed. The spectral evidence support the binding of HSA to the Cu(II) complex but as the absorbance spectra does not record any shift in the wavelength, the reduced binding or low binding affinity for HSA is concluded . The rational design of compounds with reduced albumin binding has been limited due to lack of binding data to albumin active sites. These results are important in understanding the

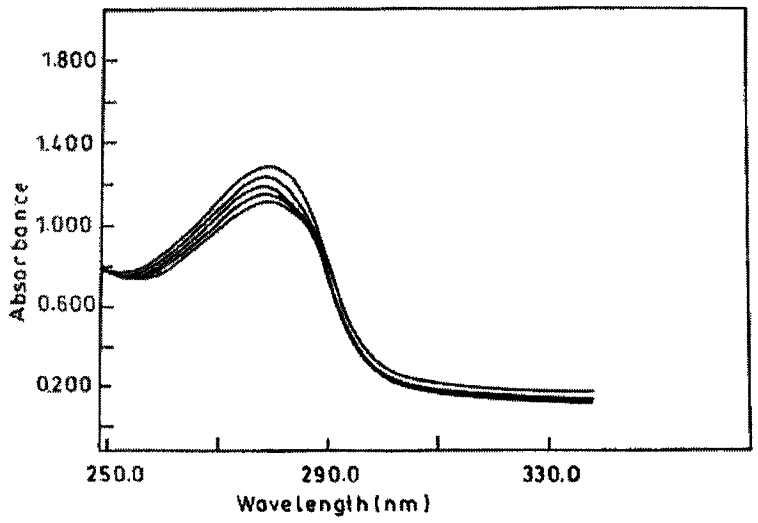

**Fig. 6:** Electronic spectra of the Cu(II) complex in the presence of HSA with respect to time at 0.5 × $10^{-5}$ mol $dm^{-3}$.

structure affinity relationship of metal complex-albumin interaction /9/.

The rate constants $k_{obs}$ were determined using the least square regression method (Fig. 7, 8). The plot of $k_{obs}$ versus [HSA] is linear suggesting pseudo first order reaction kinetics. The following rate law holds good:

$k_{obs} = k_1k_2[HSA]/[k_{-1} + k_2]$

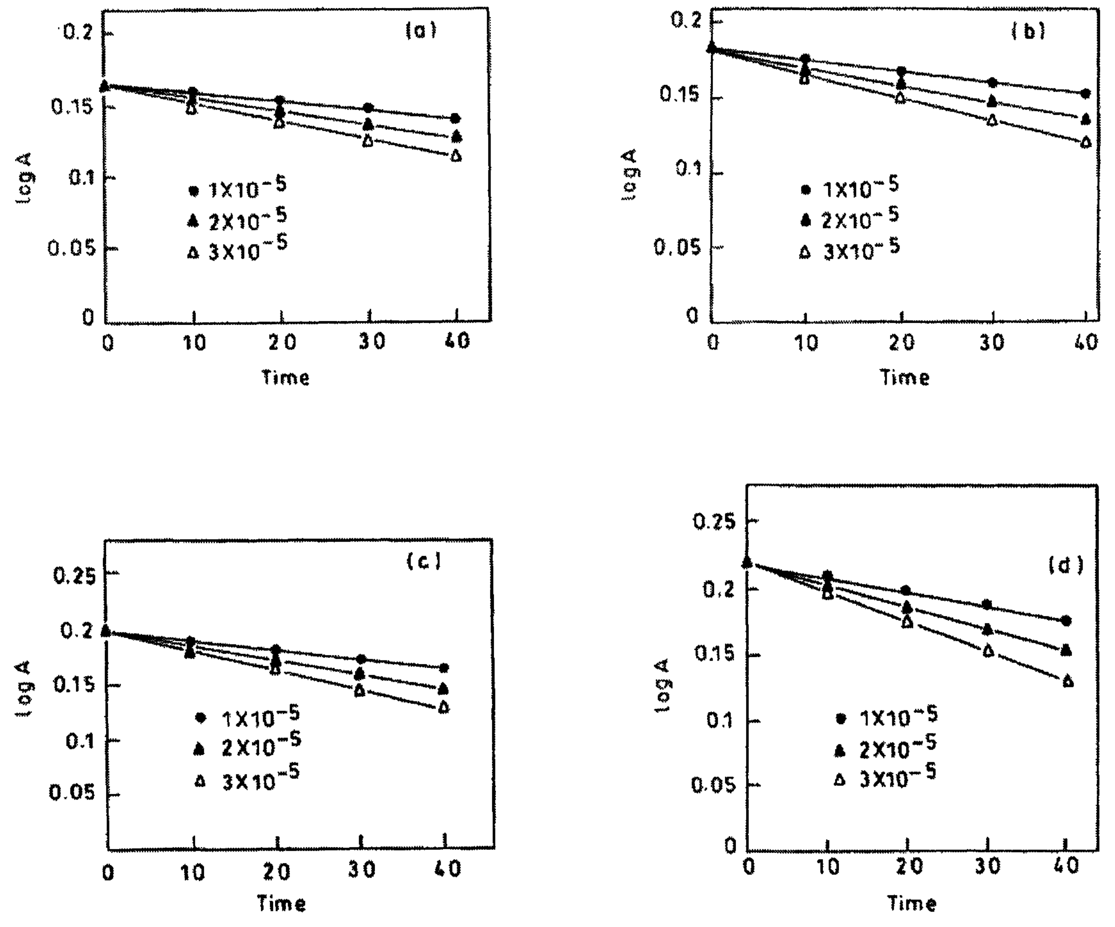

Fig. 7: (a) Plot of logA versus time at varying concentrations (c=1-3 x $10^{-5}$ mM) of HSA at 6.0 pH

(b) Plot of logA versus time at varying concentrations (c=1-3 x $10^{-5}$ mM) of HSA at 6.5 pH

(c) Plot of logA versus time at varying concentrations (c=1-3 x $10^{-5}$ mM) of HSA at 7.0 pH

(d) Plot of logA versus time at varying concentrations (c=1-3 x $10^{-5}$ mM) of HSA at 7.4 pH

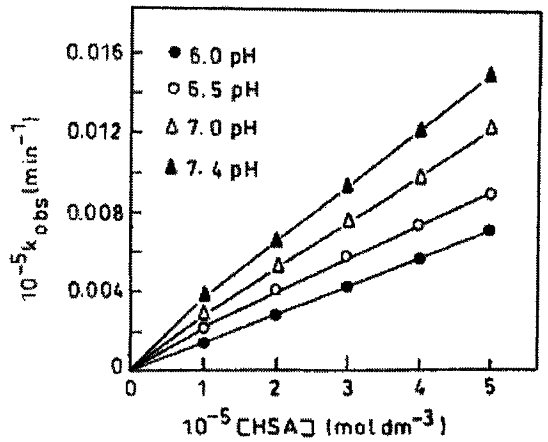

**Fig. 8:** Plot of $k_{obs}$ versus HSA (protein) at varying concentrations (c = 1-5 × $10^{-5}$ mM) and different pH.

### *pH Profile*

We have also studied the reaction kinetics of the Cu(II) complex in the presence of HSA at different pH values and consequent changes in absorption spectra were observed. There is a negligible change in the absorbance spectra in 6.0 - 7.4 pH range. At pH 7.4 the absorption spectra does not show any shift although a high maxima is observed indicating that the interaction between the complex and serum albumin is weak. However as the pH values decreases, the intensity of the absorption peak at 278 nm ($\lambda_{max}$ of HSA) decreases, indicating that the coordination of the Cu(II) complex to serum protein may take place through imidazole side chains of surface exposed histidine of protein /33/.

The plots of $k_{obs}$ *versus* HSA at different pH's give a straight line suggesting that all the reactions are of pseudo-first order type (Fig. 9).

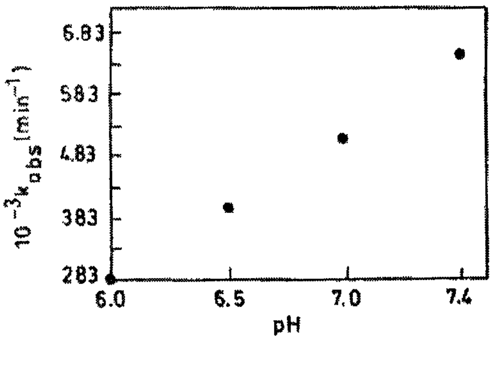

**Fig. 9:** pH-rate profiles of Cu(II) complex bound with HSA.

### ACKNOWLEDGMENTS:

We are grateful to Dr. Sudha Srivastava, TIFR Mumbai. Special thanks are due to Dr. Sartaj Tabassum, Department of Chemistry, AMU Aligarh for providing cyclic voltammetry facilities. We are also thankful to the CDRI Lucknow for providing C,H,N and IR facilities.

### REFERENCES

1. W. Bal, J. Christodolou, P. J. Sadler and A. Tucker, *J. Inorg. Biochem*, **70**, 33 (1998).
2. P. J. Sadler and J. H. Viles, *Inorg. Biochem.*, **35**, 4490 (1996).
3. M. W. Foster and J. A. Cowan, *J. Am. Chem. Soc.*, **121**, 4093 (1999).
4. S. Adhikari, R. Joshi and C. Gopinathan, *Biochimica et Biophysica Acta*, **1380**, 109 (1998).
5. G. C. Roberts, *Curr. Opin. Biotechnol.*, **10**, 42 (1999).
6. R. E. Hubbard, *Curr. Opin. Biotechnol.*, **8**, 696 (1997).
7. W. N. Hunter, *Parasitology* **114** Suppl. S 17 (1997).
8. M. L. Kleinberg and L. A. Wanke, *Am. J. Health Syst. Pharm.* **52**, 1323, 1341 (1995).
9. H. Mao, P. J. Hajduk, R. Craig, R. Bell, T. Borre and S. W. Fesik, *J. Am. Chem. Soc.*, **123**, 10429 (2001).
10. T. Peters, *All about Albumin: Biochemistry, Genetics and Medical Application*, Academic Press: San Diego; 1998, 1996.
11. E. T. Olejniczak, R. P. Meadows, H. Wang, M. Cai, D. G. Nettesheim and S. W. Fesik, *J. Am. Chem. Soc.*, **121**, 9249 (1999).
12. M. A. Navia and M. A. Murcko, *Curr. Opin. Struct. Biol.*, **2**, 202 (1992).
13. J. Greer, J. W. Erickson, J. J. Baldwin and M. D. Varney, *J. Med. Chem.* **37**, 1035 (1994).
14. B. Nerli, D. Romanin and G. Pico, *Chem.-Biol. Interact*, **104**, 179 (1997).
15. L. M. Russ, *J. Org. Chem.* **34**, 615 (1969).
16. N. Diaz, D. Suarez and T. L. Sordo, *J. Am. Chem. Soc.*, **122**, 6710 (2000).
17. B. Mohani, S. Tabassum and F. Arjmand, *Transition Metal Chem.* **27**, 776 (2002).
18. G. Wilkinson, R. D. Gillard and J. A. McCleverty, *Coordination Chemistry*, **2**, 50 (1987).
19. M. C. Jain, A. K. Srivastava and P. C. Jain, *Inorg. Chim. Acta*, **23**, 199 (1977).
20. A. A. Tak, F. Arjmand and S. Tabassum, *Transition Metal Chem.* **27**, 741 (2002).
21. K. Sakata, M. Hashimoto, T. Kashiwamura and A. Tsuge, Synth. *React. Inorg. Met. Org. Chem.*, **27**, 797 (1997).
22. F. Athar, F. Arjmand and S. Tabassum , *Transition Metal Chem.* **26**, 574 (2001).
23. K. K. Nanda, A. W. Addison, N. Paterson, E. Sinn, L. K. Thompson and U. Sakaguchi, *Inorg. Chem.* **37**, 1028 (1998).
24. T. Pintauer, J. Qiu, G. Kickelbick and K. Matyjaszewski, *Inorg. Chem.* **40**, 2818 (2001).
25. A. K. Singh, B. K. Puri and R. K. Rawllay, *Indian J. Chem.* **27A**, 430 (1988).
26. S. Chawala, *Indian J. Chem.*, **37A**, 904 (1998).

27. F. Athar, F. Arjmand and S. Tabassum, *Transition Metal Chem.* **26**, 426 (2001).
28. S. Sailaja, M. R. Reddy, K .M. Raju and K. H. Reddy, *Indian J. Chem.* **38A**, 156 (1999).
29. A. B. P. Lever, *Inorganic Electronic Spectroscopy,* Elsevier, Amsterdam, 1968; p. 343.
30. K. K. Nanda, R. Das, K. Venkatsubramanian, P. Paul and K. Nag, *J. Chem. Soc. Dalton Trans* **1993**, 2515.
31. A. J. Bard and L. R. Faulkner, *Electrochemical Methods*, Wiley, New York, 1980; p, 219.
32. C. Giacomelli, K. Ckliess, D. Galato, F. S. Miranda and A. Spinelli, *J. Braz. Chem. Soc.*, **13**, 332 (2002).
33. M. A. Fazal, B. C. Roy, S. Sun, S. Mallik and K. R. Rodgers, *J. Am. Chem. Soc.* **123**, 6283 (2001).

